# Finite Element Parametric Design of Hallux Valgus Orthosis Based on Orthogonal Analysis

**DOI:** 10.1111/os.13862

**Published:** 2023-09-05

**Authors:** Zhi Tang, Yifei Wu, Wenlan Bao, Xiaoyan Chen, Die Zhang, Alexander Nikolaevich Korotkov, Weiming Zheng, Song Gu

**Affiliations:** ^1^ College of Mechanical Engineering Donghua University Shanghai China; ^2^ Institute of IT, Mechanical Engineering and Motor Vehicles T.F. Gorbachev Kuzbass State Technical University Kemerovo Russia; ^3^ Trauma Center, Shanghai General Hospital Shanghai Jiao Tong University School of Medicine Shanghai China

**Keywords:** Bunion, Finite Element Simulation, Orthogonal Experimental Design, Orthosis

## Abstract

**Objective:**

To design appropriate orthosis for hallux valgus, a difficult foot condition that affects a quarter of the body's bones, we need to clarify the numerical biomechanical features, which have not been established in previous biomechanical studies. Therefore, we constructed a finite element model of the bunion foot to investigate the orthopaedic force compensation mechanism.

**Methods:**

A patient with moderate hallux valgus was recruited. CT imaging data in DICOM format were extracted for three‐dimensional foot model reconstruction. In conjunction with the need for rapid design of bunion orthosis, a metatarsal force application sizing method based on an orthogonal test design was investigated. The orthogonal test design was used to obtain the hallux valgus angle (HVA) and the inter metatarsal angle (IMA) data for different force combinations. Based on the extreme difference analysis and analysis of variance of the test results, the influence of different force combinations on the bunion angle was quickly determined.

**Results:**

The results showed that the stress concentration occurred mainly in the first metatarsal bone. The distribution trend was in the medial and lateral middle of the bone and gradually decreased to the dorsal base of the bone body. The greatest stress occurs in the cartilage between the phalanges and metatarsals. In 25 groups of simulation experiments, HVA was reduced from 27.7° to 13°, and IMA was reduced from 12.5° to 7.3°.

**Conclusion:**

Applying detailed orthopaedic force collocation to the first metatarsal column can effectively restore the mechanics and kinematics of hallux valgus, and provide a reference for the treatment of bunion valgus and the design of orthopaedic devices.

## Graphical abstract

A patient with moderate hallux valgus was recruited. CT imaging data in the DICOM format were extracted for 3D foot model reconstruction. In conjunction with the need for rapid design of bunion orthoses, a metatarsal force application sizing method based on an orthogonal test design was investigated. The orthogonal test design is used to obtain hallux valgus angle (HVA) and the inter metatarsal angle (IMA) data for different force combinations. Based on the extreme difference analysis and analysis of variance of the test results, the influence of different force combinations on the bunion angle was quickly determined. The results showed that the stress concentration occurred mainly in the first metatarsal bone. The distribution trend was in the medial and lateral middle of the bone and gradually decreased to the dorsal base of the bone body. The greatest stress occurs in the cartilage between the phalanges and metatarsals. In 25 groups of simulation experiments, HVA was reduced from 27.7° to 13°, and IMA was reduced from 12.5° to 7.3°. Applying detailed orthopaedic force collocation to the first metatarsal column can effectively restore the mechanics and kinematics of hallux valgus and provide reference for the treatment of bunion valgus and the design of orthopaedic devices.

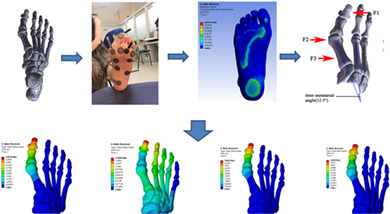

## Introduction

A bunion is a foot deformity characterized by a deviation of the bunion and a widening of the first and second intermetatarsal angles.^1^ As the medial side of the first metatarsal is often externally compressed by the pressure of footwear, a prominent bump is gradually formed, often accompanied by a cyst, called a bunion, which can cause considerable pain to the patient.^2^ Previous biomechanical studies have evaluated metatarsal stress, the valgus angle, and phalangeal displacement after bunion osteotomy, with some studies specifying ligamentous laxity in the foot causing valgus.^3^ The cause of bunions remains controversial, but overactivity of the metatarsals due to instability of the bunion and bunion muscles near the first metatarsal row is considered the root cause of bunions.^4^ There are various methods of metatarsal treatment, mainly involving shifting the position of the first metatarsal and changing the length of the first metatarsal to change the pressure distribution in the forefoot and return it to a normal stress state, thus correcting the bunion deformity.

Studies in the year of 2011 have found that the use of bunion orthosis is effective in relieving patients’ pain by applying external forces to shift the position of the metatarsal bones to improve the position of the first metatarsal row and prevent pressure.^5^ Controlling the pronation or eversion by supporting the medial longitudinal arch by orthosis^6^, ^7^, ^8^ may slow down the deterioration process and supplement surgical outcomes to reduce recurrence. However, as current orthosis designs rely on subjective perceptions^9^, ^10^, ^11^ and do not take into account internal biomechanical influences, they are not as effective as they could be. The reverse‐engineered orthosis has a contact surface that fits perfectly to the skin surface, while the internal skeletal anomalies lead to local stress concentrations that can cause redness and pain to the soft tissue surface. Finite elements, because of their ability to model physiological states under different conditions, have been increasingly used in recent years to explore the biomechanical mechanisms of disease and the simulation of treatment modalities. To better match real‐life situations, researchers used a finite element foot model reconstructed from normal participants to simulate the potential relationship between altered ligament‐to‐joint loading on the foot and bunions^12^ and constructed a complete foot model for quantitative assessment of the stresses acting on the foot in an upright position.^13^ The boundary surfaces of the bones and skin of the foot were then analyzed using the finite element analysis software ANSYS. An inverse finite element analysis was performed using an axisymmetric model, and the thickness of the orthopaedic insole was adjusted.^14^ The potential of finite element simulation for applications in the direction of foot biomechanics is evident. In engineering design, the combination of forces exerted by orthosis is often based on physician experience or subjective patient perception, with large errors detrimental to orthotic outcomes. In the early stages of orthosis design, it is necessary to obtain fast and accurate force application combinations that meet the requirements. Orthogonal tests^15^, ^16^ can attain training samples through fewer sample points and reduce the design complexity. This study used finite element simulation to analyze the stress distribution and joint angle variation in the first metatarsal region under the detailed contact surface. It also applied the orthogonal test method to explore the combination and matching of orthotic forces and to investigate the mechanism of orthotic force compensation.

The purpose of this study was to: (i) investigate the biomechanical characteristics of normal and hallux valgus feet under different load conditions; (ii) compare the effects of combination modes of several forces on the load and distribution of the toes and metatarsus; and (iii) provide a reasonable initial value for the optimization iteration of the scheme and a basis for the design of the hallux valgus orthosis by using a simple and low‐frequency experimental method.

## Materials and Methods

### Finite Element Model Construction

**FIGURE 1:**
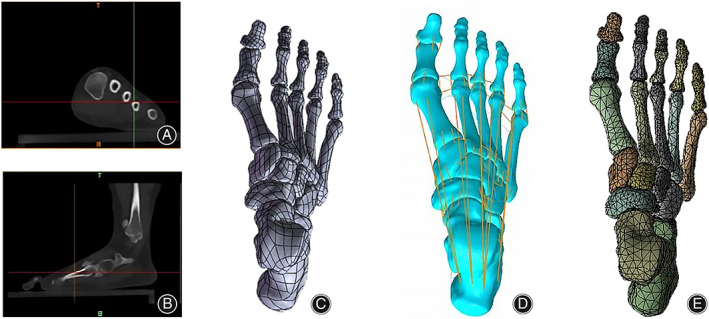
The workflow of a subject‐specific musculoskeletal dynamic modeling: (A) Coronal view of CT. (B) Sagittal view of CT. (C) Reconstructed bone tissue. (D) Simulation of the ligament‐connecting units. (E) Mesh creation for element analysis.

A 22‐year‐old female patient with a moderate bunion, body mass of 51 kg, height of 160 cm, hallux valgus angle (HVA) of 27.7°, and intermetatarsal angle (IMA) of 12.5°, was selected for CT scanning of the foot. The entire foot below the ankle joint and the initial angle were measured. The CT images were converted into DICOM format and imported into MIMICS V21.0 software (Materialize, Leuven, Belgium), where the toe, tarsal and metatarsal bones, as well as ligaments and muscles, were depicted by the anatomical structure of the human body. The skeletal model was then smoothed and wrapped into a Nubers surface model in Geomagic Wrap 2017 software (3D Systems, Rock Hill, South Carolina, USA), followed by the alignment of the skeletal positions in SOLIDWORKS 2016 software (Dassault Systèmes, Vélizy‐Villacoublay, France) and the drawing of the cartilage. 1qThe procedure for processing the model is shown in Figure 1. The exact angle value was calculated automatically using the offset angle tool in ANSYS 16.0 software (ANSYS, Canonsburg, Pennsylvania, USA).

### Material Properties and Boundary Conditions

The skin material of the foot was simulated with an isotropic and incompressible linear elastic material with a density of 1100 kg/m^3^,^17^ an elastic modulus of 0.15 MPa, and a Poisson's ratio of 0.46. The skeletal structures, such as the pelvis, spine, and lower limb bones, were simulated with a rigid material with a density of 1700 kg/m^3^ and a Poisson ratio of 0.3. The muscle soft tissue material is very viscoelastic and nonlinear.^18^ The Mooney–Rivlin model of hyperelasticity in LS_dyna software was used to simulate the strain energy function:

$$U=C_{10}\left(I_{1}-3\right)+C_{01}\left(I_{2}-3\right)+\frac{1}{D_{1}}\left(J^{\mathrm{el}}-1\right){,}^{2}$$

where $C_{10}$ is the shear modulus, taken as 1.65 KPa;

$C_{01}$ is the shear modulus, taken as 3.35 KPa;

$D_{1}$ is the bulk modulus, taken as 3.65 KPa;

$J^{\mathrm{el}}$ is the elastic volume ratio.

Here, $I_{1}$ and $I_{2}$ are the first and the second invariants of the Kirschgren equivariant form tensor,

$$I_{1}={\lambda_{1}}^{2}+{\lambda_{2}}^{2}+{\lambda_{3}}^{2}$$

and

$$I_{2}={\lambda_{1}}^{2}{\lambda_{2}}^{2}+{\lambda_{2}}^{2}{\lambda_{3}}^{2}+{\lambda_{1}}^{2}{\lambda_{3}}^{2},$$

of which $\lambda_{1}=1+\gamma_{i}$, $\lambda_{1}$ is the principal elongation, and $\gamma_{i}$ is the principal strain.

### Plantar Pressure Measurement and Validation

**FIGURE 2:**
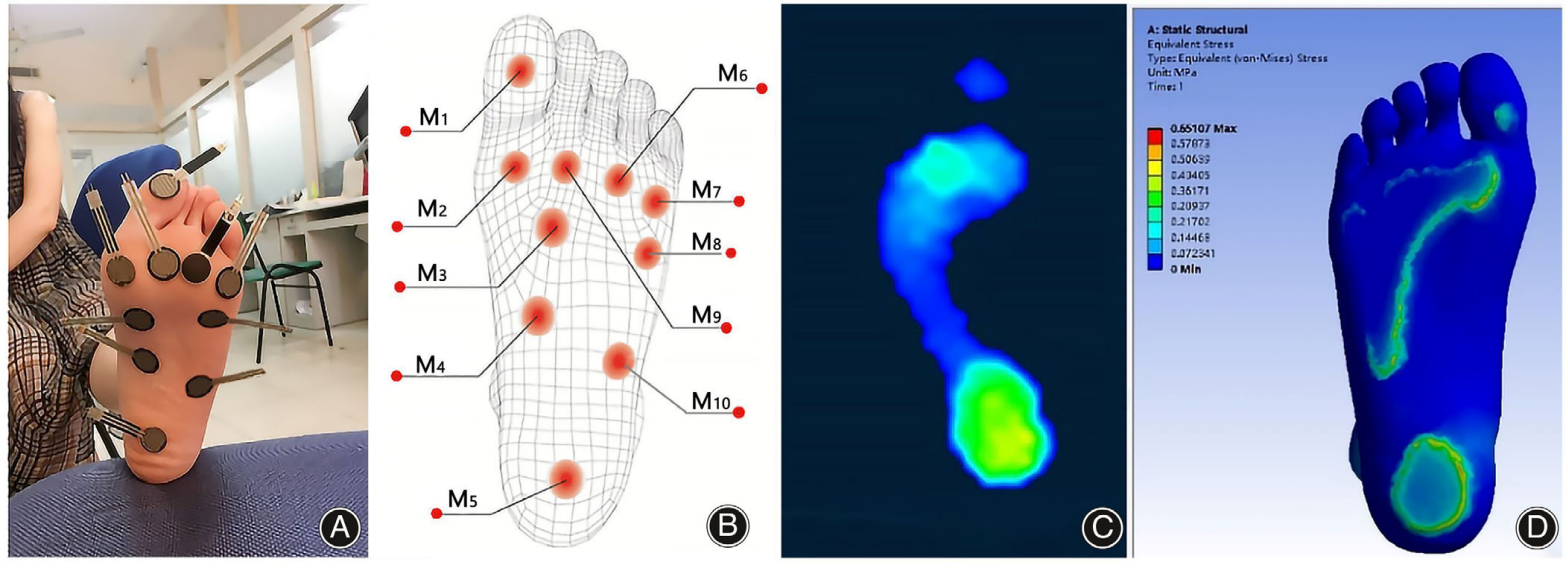
Accuracy verification of simulation model: (A) pressure test experiment collection’ (B) plantar pressure plate position diagram; (C) test result; and (D) finite element simulation result.

During this experiment, the Mangold‐10 wireless Bluetooth poly synthesizer was used to collect plantar pressure, and the electrode pads were placed in direct contact with the skin to obtain signals, as shown in Figure 2A,B. During the test, the subject remained standing position, and the test data was collected continuously for 1 min. The results of the body pressure test for plantar pressure are shown in Figure 2C. The pressure of the simulated foot model was limited to only moving in the vertical direction to ensure that the angle between the foot and the ground was consistent with the test. Before the simulation began, the foot was placed as close to the bottom plate as possible, without penetrating it, to simulate the slow fall of the foot. Figure 2D shows the simulation results for the human foot sole model. The simulation results of the body pressure distribution cloud for the rigid sole matched well with the test results, with the maximum pressure values located under the heel and forefoot and the body pressure values gradually decreasing outwards along the heel. According to the simulation results, the simulated maximum pressure values were smaller compared to the test values, probably due to the asymmetry of the pressure on the foot when the human body is standing, which leads to an asymmetric distribution of the actual body pressure measured in the test; the presence of the body pressure sensor increases the stiffness of the contact. In summary, the errors in the simulated results for each indicator of the body pressure distribution were small, indicating that the simulated results for the human foot sole model have a high degree of confidence, and the accuracy of the model has been verified.

### Finite Element Indicators

To evaluate the effectiveness of the bunion correction, we used two main indicators: the HVA and the IMA. The HVA is the angle between the longitudinal axis of the first metatarsal and the proximal phalanx of the hallux, and the IMA is the angle between the longitudinal axes of the first and second metatarsals. The smaller the HVA and IMA, the better the correction effect. We also calculated the stress distribution and deformation of the foot sole model under different force conditions using ANSYS software. The stress and deformation reflect the biomechanical changes of the foot structure after correction and can provide guidance for optimizing the design of corrective devices.

### Orthogonal Test Design and Analysis

Orthogonal design refers to the use of orthogonal tables to rationalize and scientifically analyze multifactor tests, using fewer tests to obtain more accurate results. The orthogonal table uses the number of factors and the level of factors in the test and is arranged according to the principle of orthogonality, which has the characteristics of neat comparability and balanced matching. The orthogonal table can be expressed as $L_{n}\left(r^{m}\right)$, where L represents the orthogonal table code, n is the number of trials, r is the number of levels, and m is the number of factors involved in the trial. Mild bunion foot: 15° < HVA < 20° and/or 9° < IMA < 11°. Moderate bunion foot: 20° < HVA < 40° and/or 11° < IMA < 16°. Severe bunion foot: HVA > 40°, IMA > 20°.^19^

**FIGURE 3:**
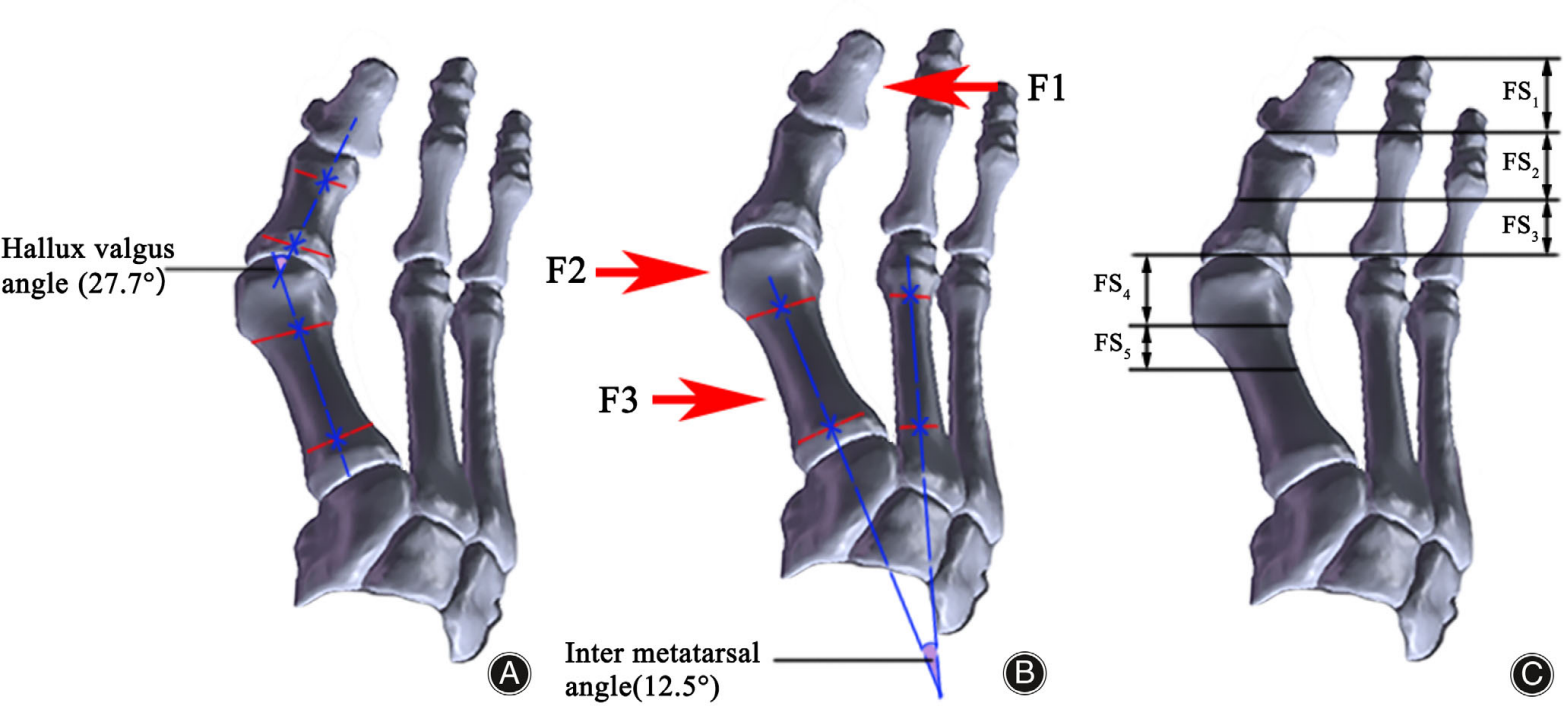
Diagram of the position of orthotic force application: (A) the method of measuring hallux valgus angle (HVA); (B) direction of orthotic force application; and (C) refining the contact surfaces of the orthotic forces in the F1 and F2 directions.

The currently accepted forces for the correction of bunions are applied from three main positions, as shown in Figure 3: F1 from the distal phalanx of the first bunion to the medial side of the foot, F2 from the area at the metatarsophalangeal joint bunion to the lateral side of the foot, and F3 from the proximal end of the first metatarsal to the medial side of the foot. This paper has a refined contact surface, divided into FS_1_–FS_5_. From the combined simulation analysis of metatarsal force application, the key forces that affect the metatarsal force can be obtained, and the application locations can be subdivided into groups.

The orthotic range of forces under the three‐point force orthotic principle is determined by applying moments F1, F2, and F3 to the metatarsophalangeal joint of the foot to provide the initial range of moments for the orthotic optimization of the orthotic forces below. The HVA and IMA were also recorded, and the stress distribution was observed. F1 was loaded from 200 N to 700 N in single increments of 100 N (fix F2 = 300 N, F3 = 400 N); F2 applied pressure values from 100 N to 600 N in single increments of 100 N (fix F1 = 500 N, F3 = 400 N), and F3 applied pressure from 300 N to 800 N in single increments of 100 N (fix F1 = 500 N, F2 = 300 N).

A polar difference analysis is presented in Table 1 to explore the degree of influence of FS_1_–FS_5_ on the results from the HVA perspective and the IMA perspective, respectively. By analyzing the magnitude of the extreme differences between the factors, the degree of influence of the different factors on the test results can be obtained. The larger the polar difference, the greater the influence of the factor on the angle, the most dominant factor. Conversely, it means that the factor has a small effect on the drag coefficient and is a non‐dominant factor.

**Table 1:** The orthogonal table and the corresponding FE predict the hallux valgus angle (HVA) *versus* the inter metatarsal angle (IMA) for the first metatarsal column.

Trial number	Design factor					FE predicated actor (°)		von Mises
	FS_1_	FS_2_	FS_3_	FS_4_	FS_5_	IMA	HVA	Stress
1	150	50	250	100	100	12.97238	13.13862	224.79
2	150	100	150	250	300	7.3019	27.7851	227.97
3	150	150	250	150	250	12.233	18.503	231.18
4	150	200	100	300	200	9.555	25.749	234.4
5	150	250	200	200	150	13.5522	17.4008	245.09
6	200	50	250	250	200	10.9066	22.3374	298.66
7	200	100	100	150	150	11.569	21.294	301.83
8	200	150	200	300	100	12.309	21.152	305.02
9	200	200	50	200	300	10.07	28.212	308.23
10	200	250	150	100	250	13.911	20.02	311.46
11	250	50	200	150	300	11.3131	24.9089	372.53
12	250	100	50	300	250	8.198	32.592	375.69
13	250	150	150	200	200	12.679	23.761	378.88
14	250	200	250	100	150	16.28	15.808	382.08
15	250	250	100	250	100	13.9826	22.6734	385.29
16	300	50	150	300	150	11.3944	27.5526	446.4
17	300	100	250	200	100	11.3944	23.2016	449.57
18	300	150	100	100	300	13.05	26.368	452.74
19	300	200	200	250	250	13.75	26.266	455.93
20	300	250	50	150	200	14.34	25.295	459.13
21	350	50	200	200	250	13.026	27.312	520.27
22	350	100	200	100	200	15.4178	22.1572	523.43
23	350	150	50	250	150	13.12157	29.02143	526.6
24	350	200	150	150	100	16.7092	21.0828	529.79
25	350	250	250	300	300	14.81	28.764	532.99

Abbreviation: FE, finite element.

### Refinement of Contact Surface Design

Although the foot has a smooth curved surface between the toes, when patients wear commercially available orthosis, the internal bony anomalies, especially at FS_2_, lead to partial pressure concentrations on the skin surface, making them prone to local pressure pain and skin abrasion over prolonged wear.

Based on the conclusions of the metatarsal force application combination simulation analysis, the key forces affecting the metatarsal force are F1 and F2. When F1 force is applied, the division selects the outer end surface of the thumb tip in three parts: S1 is 0–15 mm in length from the tip position, S2 is 15–30 mm from the tip position, and S3 is 30–45 mm from the tip position.

For five different areas, such as FS_1_, FS_2_, FS_3_, FS_4_, and FS_5_, the magnitude of the applied force is used as factors, according to the five levels given in Table 1 using. The experimental design optimizes the extraction conditions, using the proposed level method, to obtain a combined scheme table of 25*5. The table design and the levels of each factor are shown in Table 1, and the maximum stress, maximum displacement, IMA and HVA are calculated at this point. When $K_{i}$ indicates that the level number of a given test factor is $i\left(\;i=1,2,\cdots ,5\right)$, the sum of the results corresponding to $k_{i}=K_{i}/5$ denotes the arithmetic mean of the resistance coefficient obtained when a factor is taken at level i.

**FIGURE 4:**
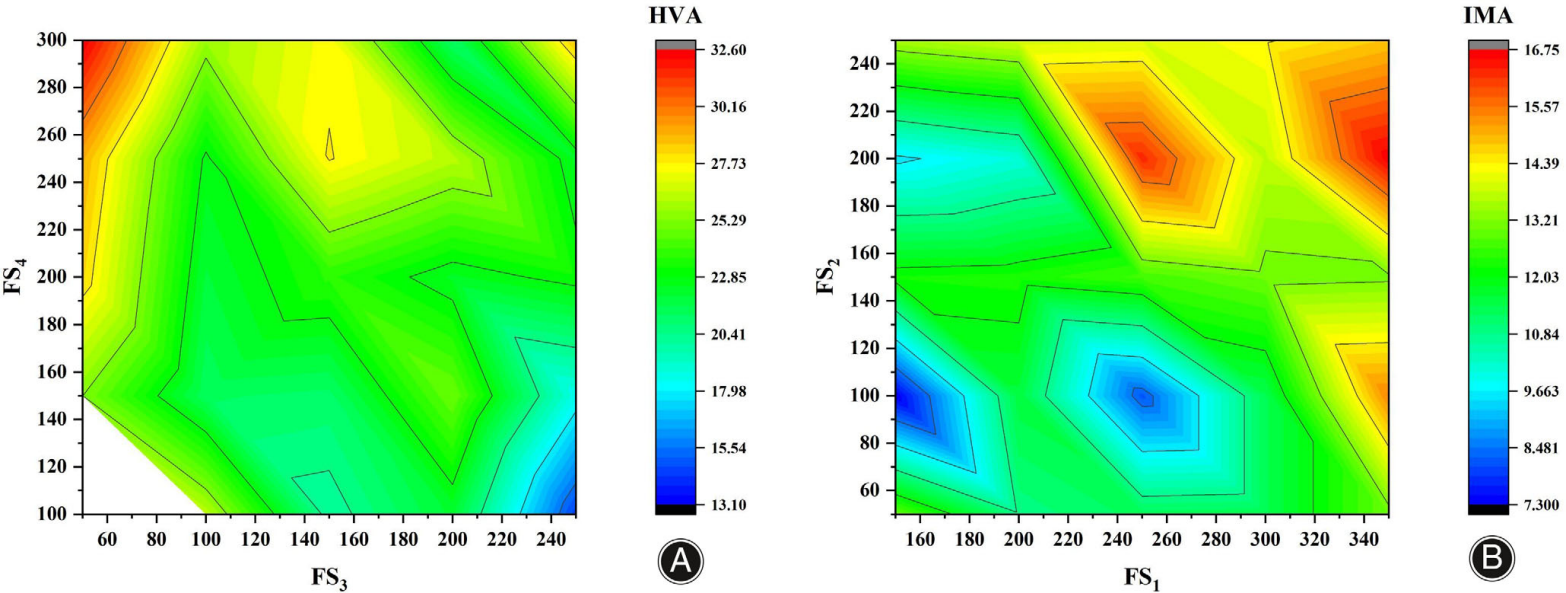
A plot of HVA angle versus IMA angle. (A) HVA angular relationship diagram; (B) IMA angular relationship diagram.

The analysis of the extreme difference results from the HVA perspective, as shown in Table 2. Since the order of polar differences is $R_{{\mathrm{FS}}_{3}}>R_{{\mathrm{FS}}_{4}}>R_{{\mathrm{FS}}_{5}}>R_{{\mathrm{FS}}_{1}}>R_{{\mathrm{FS}}_{2}}$, the order of influence for the HVA from primary to secondary is FS_3_, FS_4_, FS_5_, FS_1_, and FS_2_. The two influential factors FS_3_ and FS_4_ were selected to show the relationship with the HVA, as shown in Figure 4A. The IMA from primary to secondary is FS_1_, FS_2_, FS_4_, FS_5_ and FS_3_. The two influential factors FS_1_ and FS_2_ were selected to show the relationship with the IMA, as shown in Figure 4B.

**Table 2:** Hallux valgus angle (HVA) and inter metatarsal angle (IMA) angular extreme variance analysis results.

	Category	FS_1_	FS_2_	FS_3_	FS_4_	FS_5_
HVA	Extreme difference R	5.22	2.58	8.49	7.66	6.96
	Primary secondary	FS_3_	FS_4_	FS_5_	FS_1_	FS_2_
IMA	Extreme difference R	3.49	3.34	1.80	3.07	2.16
	Primary secondary	FS_1_	FS_2_	FS_4_	FS_5_	FS_3_

Combining the relationship between the superior levels of the different factors and the HVA, if there is a positive contribution, e.g., the maximum HVA, the corresponding factor level is selected, that is, the choice of the columns in $K_{i}$ or $k_{i}$ is the corresponding maximum level in each column. However, for bunion metatarsals, the smaller the HVA the better, so picking each $K_{i}$ or $k_{i}$ at the level corresponding to the smallest value is the optimal solution for correcting the HVA. Therefore, FS_1_ = 150 N, FS_2_ = 100 N, FS_3_ = 250 N, FS_4_ = 100 N, and FS_5_ = 100 N.

**FIGURE 5:**
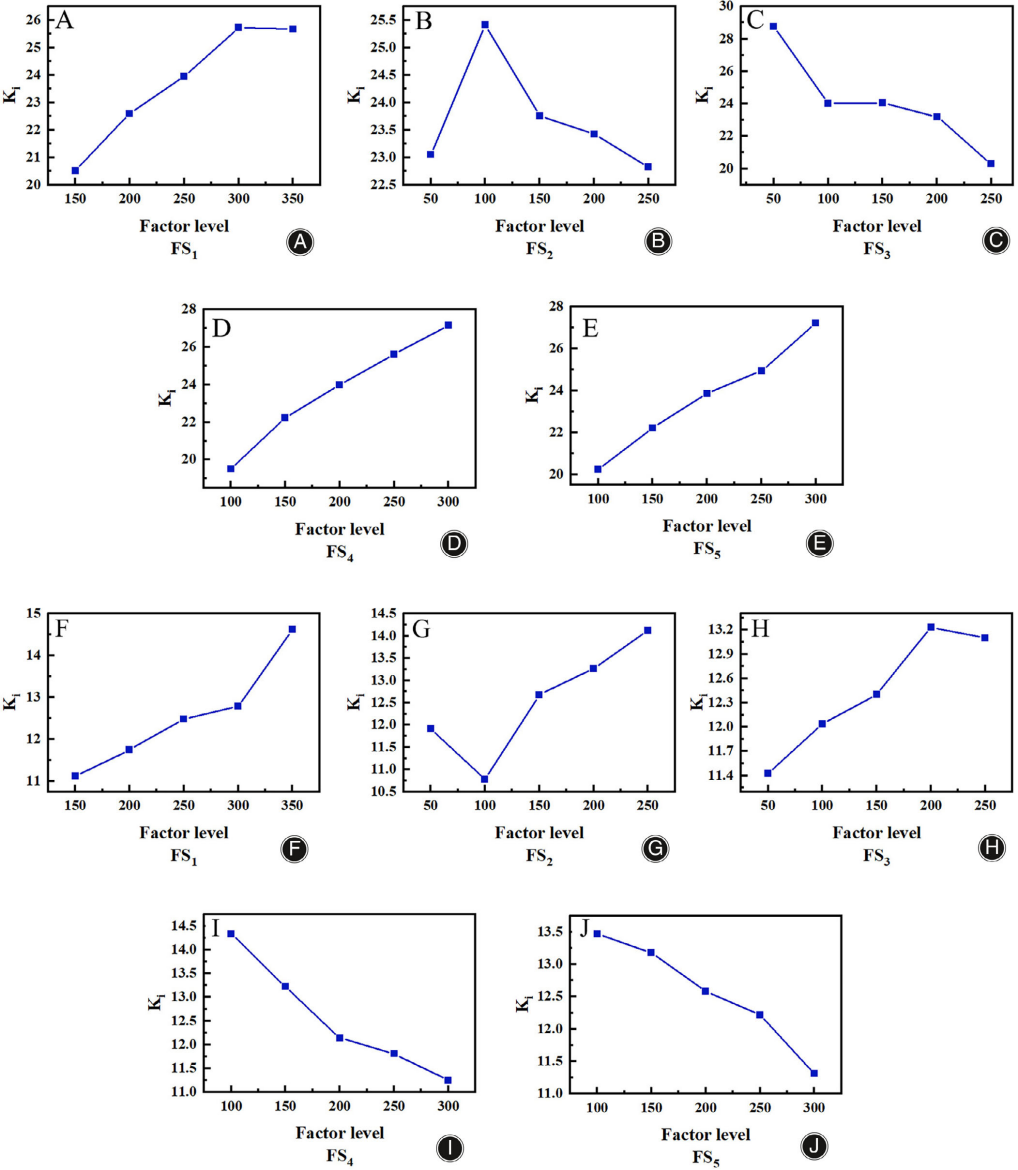
(A) Horizontal‐mean trend graph of FS1 factor for HVA; (B) Horizontal‐mean trend graph of FS2 factor for HVA; (C) Horizontal‐mean trend graph of FS3 factor for HVA; (D) Horizontal‐mean trend graph of FS4 factor for HVA; (E) Horizontal‐mean trend graph of FS5 factor for HVA; (F) Horizontal‐mean trend graph of FS1 factor for IMA; (G) Horizontal‐mean trend graph of FS2 factor for IMA; (H) Horizontal‐mean trend graph of FS3 factor for IMA; (I) Horizontal‐mean trend graph of FS4 factor for IMA; (J) Horizontal‐mean trend graph of FS5 factor for IMA.

As can be seen from Figure 5, the HVA gradually increases for FS_1_, FS_4_, and FS_5_ and for FS_2_ shows a trend of increasing and then decreasing, but the overall difference is not significant, with FS_3_ having the largest fluctuating value and being the optimization focus. Combining the superior level of different factors in relation to the IMA, for bunion orthosis, the smaller the IMA, the better, so picking each $K_{i}$ or $K_{i}$ at the level corresponding to the smallest value is the optimal solution for correcting the IMA. Therefore, FS_1_ = 150 N, FS_2_ = 100 N, FS_3_ = 50 N, FS_4_ = 300 N, and FS_5_ = 300 N.

## Results

### Effects of F1, F2, and F3 on Hallux Valgus Angle, Inter Metatarsal Angle, and Stress Distribution

**FIGURE 6:**
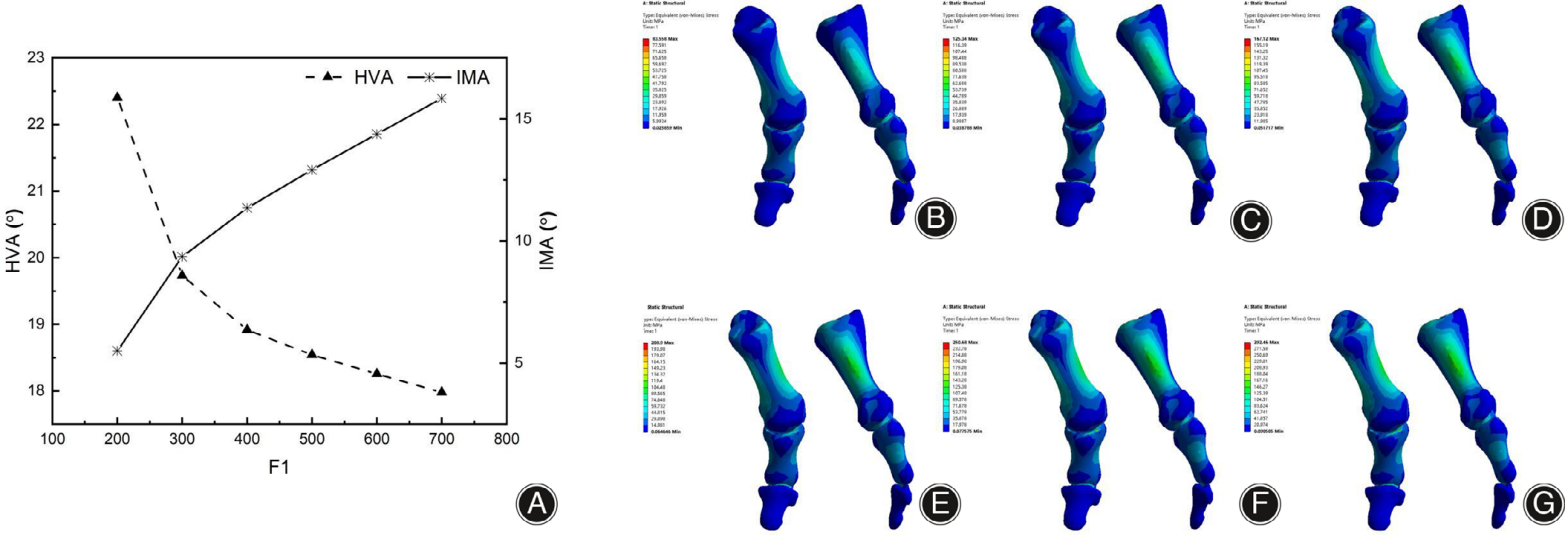
(A) HVA and IMA line charts under F1 variation; (B) stress nephogram of the first metatarsal column when F1 is 200N; (C) stress nephogram of the first metatarsal column when F1 is 300N; (D) stress nephogram of the first metatarsal column when F1 is 400N; (E) stress nephogram of the first metatarsal column when F1 is 500N; (F) stress nephogram of the first metatarsal column when F1 is 600N; (G) stress nephogram of the first metatarsal column when F1 is 700N.

Under the three‐point force orthotic principle, the maximum stress occurs in the cartilage in the middle of the proximal phalanx, and its value increases with increasing F1 force, ranging from 83.558 MPa to 292.46 MPa in the range of 200 N‐700 N (Figure 6). The pressure distribution also increases with increasing F1 force and tends to increase on both sides. The smaller the HVA, the less desirable the IMA. From 400 N onwards, the HVA stays at around 18 degrees, while the IMA increases from 11 to 15 degrees. We find that the HVA decreases as F1 increases, but continuously increasing the F1 value does not make the HVA continue to decrease; it increases instead. The IMA gradually becomes larger with increasing F1; the recommended range for F1 is 300 N–500 N.

**FIGURE 7:**
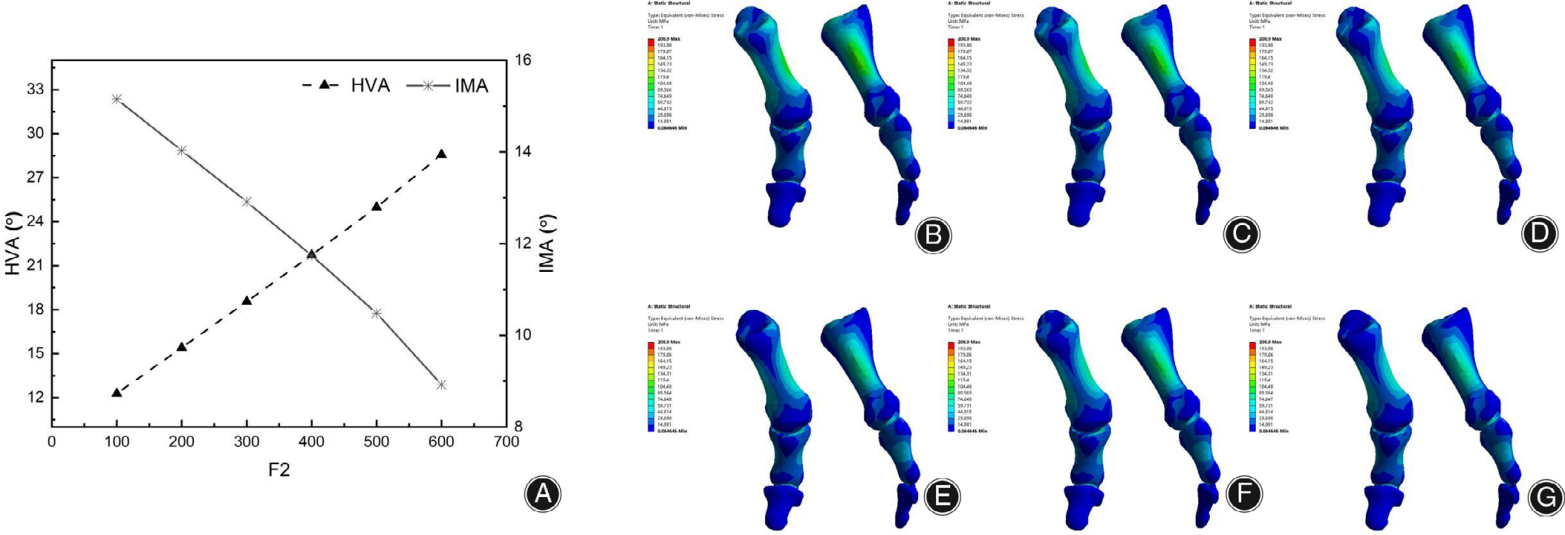
(A) HVA and IMA line charts under F2 variation; (B) stress nephogram of the first metatarsal column when F2 is 100N; (C) stress nephogram of the first metatarsal column when F2 is 200N; (D) stress nephogram of the first metatarsal column when F2 is 300N; (E) stress nephogram of the first metatarsal column when F2 is 400N; (F) stress nephogram of the first metatarsal column when F2 is 500N; (G) stress nephogram of the first metatarsal column when F2 is 600N.

As F2 increased, the stress concentration range of the cartilage in the middle of the proximal phalanx also increased. HVA increased gradually with the increase of pressure in the range of 100 N to 600 N, but HVA exceeded the healthy range when it was above 200 N. IMA gradually decreased with increasing pressure (Figure 7) and reached a healthy range after 400 N. Therefore, the recommended range for F2 is 100 N to 400 N.

**FIGURE 8:**
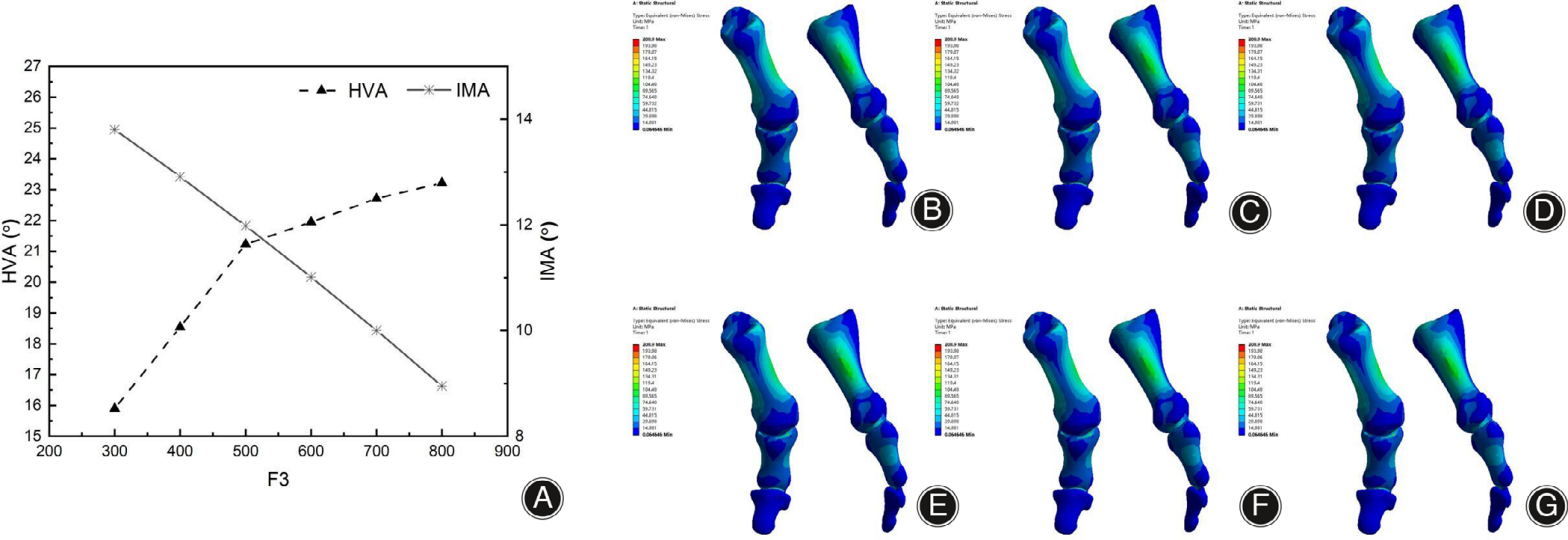
(A) HVA and IMA line charts under F3 variation; (B) stress nephogram of the first metatarsal column when F3 is 300N; (C) stress nephogram of the first metatarsal column when F3 is 400N; (D) stress nephogram of the first metatarsal column when F3 is 500N; (E) stress nephogram of the first metatarsal column when F3 is 600N; (F) stress nephogram of the first metatarsal column when F3 is 700N; (G) stress nephogram of the first metatarsal column when F3 is 800N.

The maximum stress was 208.9 MPa in the range of 300 N to 800 N, indicating that F1 was the main factor affecting the maximum stress of the proximal phalangeal bone relative to F3. The pressure distribution of the first metatarsal bone was in the range of 300 N to 800 N, gradually increasing from 8.38 MPa to 12.86 MPa, and progressively increasing with the increase of F3 force. HVA increases gradually with the increase of pressure and changes little after 500 N. Its distribution is concentrated around 22° (Figure 8). IMA decreased gradually with the increase in pressure, but the change was not large. The results showed that compared with F1 and F2, the orthotic effect of F3 was not significant, mainly due to the fixed effect, and excessive force would cause pain in patients.

### Orthogonal Test Results

**FIGURE 9:**
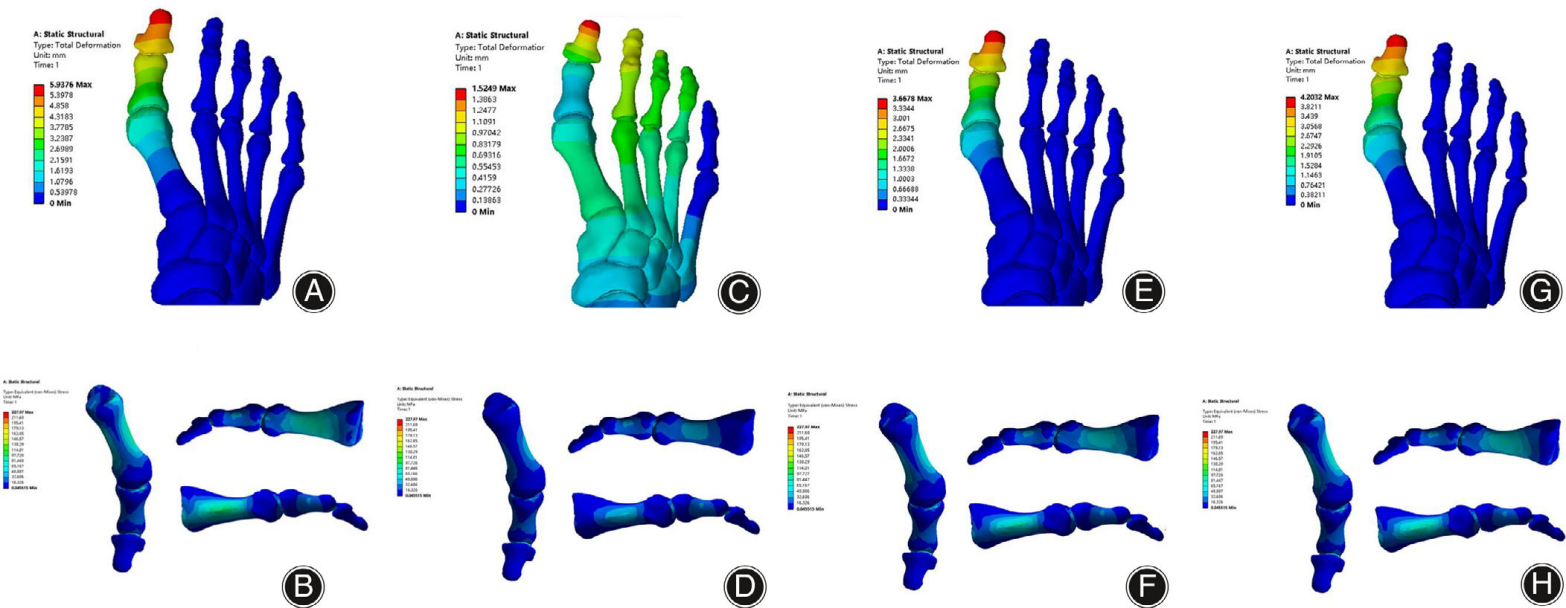
(A) The deformation nephogram of foot in Group 1. (B) The strain nephogram of the first metatarsal column in Group 1. (C) The deformation nephogram of foot in Group 2. (D) The strain nephogram of the first metatarsal column in Group 2. (E) The deformation nephogram of foot in Group 3. (F) The strain nephogram of the first metatarsal column in Group 3. (G) The deformation nephogram of foot in Group 4. (H) The strain nephogram of the first metatarsal column in Group 4.

For the optimal solution for the correction of HVA, Group 1: FS1 = 150 N, FS2 = 100 N, FS3 = 250 N, FS4 = 100 N, and FS5 = 100 N; at this time HVA = 12.669° and IMA = 13.689°; at this time the IMA is large, it is a moderate bunion, and the stress range is the largest, producing a stronger compression. The optimal solution for correcting the IMA, Group 2: FS1 = 150 N, FS2 = 100 N, FS3 = 50 N, FS4 = 300 N, and FS5 = 300 N. The simulation shows that HVA = 30.04° and IMA = 7.6°. The IMA is ideal, and the range of stress is small and comfortable, but the HVA becomes large again, and the metatarsal effect is not ideal. The two scenarios were averaged and then combined. Group 3 was FS1 = 150 N, FS2 = 100 N, FS3 = 150 N, FS4 = 200 N, and FS5 = 200 N. The simulation at this point resulted in HVA = 22.038°and IMA = 9.97°. The HVA is close to the mild bunion range, but there is still a gap with the mild bunion and further downward adjustment of the HVA. According to the data of orthogonal analysis and the trend of the mean factor level, it is evident that FS3 has the greatest influence on the HVA. Therefore, the final value of the best solution in Group 4 was taken as follows: FS1 = 150 N, FS2 = 100 N, FS3 = 200 N, FS4 = 200 N, and FS5 = 200 N. At this point, HVA = 17.49° and IMA = 10.21°; the correction effect has been achieved, and the stress range was moderate and comfortable (Figure 9). Therefore, in general, the stress is mainly concentrated on the first metatarsal in all four groups. Group 1 has a stress range that is too large, Group 2 is not suitable for large HVA scenarios, and Group 3 is the average of Group 1 and Group 2, but still not ideal. Group 4 has the best correction effect and the most appropriate stress range.

## Discussion

### Summary of Main Findings

In this study, we aimed to find the optimal combination of orthotic forces for correcting mild to moderate bunions using finite element analysis and orthogonal test design. We applied three orthogonal forces (F1, F2, and F3) to the first metatarsophalangeal joint of a bunion foot model under different magnitudes and positions. We evaluated the effects of these forces on HVA, IMA, and stress distribution. We found that F1 and F2 were the most influential factors for HVA and IMA correction, respectively. The optimal combination of forces was FS1 = 150 N, FS2 = 100 N, FS3 = 200 N, FS4 = 200 N, and FS5 = 200 N. This combination reduced the HVA from 27.7° to 17.49° and the IMA from 12.5° to 10.21°, while avoiding stress concentration.

### Biomechanical Mechanisms and Factors Involved in Bunion Correction

In the study conducted by Menz *et al*.^20^ on 172 volunteers, it was observed that a higher degree of bunion angle resulted in increased load on the bunion. This phenomenon may be due to the deformation of the bunion, which alters the propulsive function of the first metatarsophalangeal joint, causing an outward deflection of the pressure load on the foot. Our findings are consistent with this, as we have observed higher von Mises stress values in the bunion foot compared to the stress distribution in the first metatarsophalangeal joint region of the normal foot. This explains the inward tilting of the foot and the formation of keratosis in the soft tissue of the medial capsule of the first metatarsophalangeal joint that may occur clinically in bunion patients due to excessive force on the first metatarsal. Reducing the forces on the metatarsals and shifting them towards the bunion is the key to alleviating these problems. One way to alleviate these problems is to reduce the force on the distal and proximal phalanxes, which can be achieved with certain foot aids. The results indicate that this method can quickly obtain orthotic force combinations that meet requirements, improve the efficiency of analysis, and guide the design of the orthotic shape. Under the principle of three‐point force orthosis, the recommended range for F1 is 300–500 N and for F2 is 100–400 N. Therefore, the recommended value for F3 is between 300 and 500 N. It is also known that F1 has a direct, but not linear, effect on the change in the value of the HVA. An appropriate increase in the value of F2 can reduce the IMA while keeping F1 constant. In addition to the correction effect of HVA and IMA, this study also explored the design features and clinical applications of bunion orthosis. We found that bunion orthosis can produce stable corrective force on the bunion through the principle of three‐point force. We also found that bunion orthosis can reduce the foot pain and discomfort of patients and improve their quality of life and satisfaction.^5^ These results are consistent with other studies. Moreover, this study also used a foot stress model in a standing position^13^ to analyze the effect of bunion orthosis on foot pressure distribution and found that bunion orthosis can reduce the peak pressure of patients, relieve foot load, and prevent foot complications.

### Clinical Implications

Our findings also have important implications for clinical treatment indicators and osteotomy plans for bunions. Rather than solely focusing on reducing HVA, which is often used as a primary outcome measure for bunion correction, indicators should also take into account changes in IMA, which reflect the degree of metatarsal splaying. In the five different directions and positions of the moments considered in this study (FS_1_–FS_5_), for the HVA, FS_1_, FS_3_, FS_4_, and FS_5_ can explain 99.73% of the reasons for the variation and play a dominant influence on the HVA, which can explain why the HVA shows a nonlinear variation when F1 changes, as the counter‐directional forces of FS_4_ and FS_5_ play an important role in limiting lateral flexion. For the IMA, FS_1_, FS_2_, and FS_4_ produced a significant difference in the IMA, with an F‐ratio of FS_1_ = 13.228 and FS_2_ = 15.028, much greater than the F‐ratio of FS_4_ = 9.536. The force applied to the proximal medial aspect of the phalanx initially reduced the IMA somewhat, but continued increases resulted in an increase in the IMA, increasing the stress on the first metatarsal. These findings provide valuable insights into the underlying mechanisms and factors involved in bunion correction and highlight the need for a comprehensive approach to clinical treatment indicators. These results also have important implications for osteotomy plans, as the aim of metatarsal interception should not only be to facilitate the rotational repositioning of the phalanx but also to achieve external displacement of the first metatarsal to reduce the intermetatarsal angle. Moreover, the study results suggest that orthopaedic aids for bunions should not just push medially on the lateral aspect of the phalanx, as this may not effectively correct bunions and may even increase the intermetatarsal grip and worsen the condition in the long term.

### Limitations and Strengths of this Study

This study has some limitations and strengths regarding the use of finite element analysis and orthogonal test design methods for bunion correction: The limitations include simplifying the bone, ligaments, and muscles, unclear experimental conditions, lack of material properties, ligament construction methods, and bunion effects on the first metatarsal and toe joints. The strengths include reducing the number of experiments, refining the contact surface design, performing an extreme difference analysis, validating the simulation results with plantar pressure measurements, and proposing a novel force compensation mechanism.

### Conclusion

According to the stress distribution and displacement of the metatarsal bones under various combinations of horizontal forces in this model, the load‐bearing capacity of the first metatarsophalangeal joint is reduced due to ligamentous laxity of the joint and high loading pressure. Our findings provide valuable insights into the biomechanical mechanisms and factors involved in bunion correction using orthosis, as well as important implications for clinical treatment indicators and osteotomy plans for bunions. The deformation of the bunion foot can be reduced through the design of biomechanical foot aids to reduce the stress on the metatarsal bones and reduce the patient's pain, providing data to support the design of the orthosis.

## Author Contributions

Zhi Tang developed the idea for the study. Yifei Wu and Wenlan Bao designed the research and wrote the paper. Xiaoyan Chen, Die Zhang, Alexander Nikolaevich Korotkov, and Weiming Zheng performed the research and analyzed the data. Song Gu provided the data. All authors contributed to the writing and revisions.

## Funding Information

This work was funded by the National Natural Science Foundation of China (No. 51775106).

## Conflicts of Interest

The authors declare that they have no conflicts of interest.

## Ethics Statement

This study was approved by the Ethics Committee of Shanghai General Hospital (2021KY118).
